# Comparison of the Ultrasonic Tip with Multidirectional Angular Cutting Geometry with the Straight Dentition Cutting in Bone Osteotomies with the Piezoelectric Technique

**DOI:** 10.3390/dj14020091

**Published:** 2026-02-05

**Authors:** Marcelo Pigatto D’Amado, Bianca Pulino, Robert Sader, Gabriele Millesi, Florian Thieringer, Geraldo Prestes de Camargo Filho, Raphael Capelli Guerra

**Affiliations:** 1 Instituto de Ensino e Pesquisa, Hospital Sírio-Libanês, São Paulo 01308-050, SP, Brazil; 2 Department of Oral and Maxillofacial Surgery, Faculdade Israelita de Ciências da Saúde Albert Einstein, São Paulo 05652-900, SP, Brazil; 3 Department of Oral and Maxillofacial Surgery, Hospital Israelita Albert Einstein, São Paulo 05652-900, SP, Brazil; 4 Department of Craniomaxillofacial and Plastic Surgery, Klinikum der Johann Wolfgang Goethe-Universität Frankfurt, Goethe University of Frankfurt, 60325 Frankfurt am Main, Germany; 5 Department of Oral and Maxillofacial Surgery, University Hospital Vienna, 1090 Vienna, Austria; 6 Universitätsspital Basel, 4031 Basel, Switzerland; 7 College of Dental Medicine, Nova Southeastern University, Clearwater, FL 33759, USA

**Keywords:** piezoelectric saw, osteotomy, multidirectional blade, cutting time, bone temperature, orthopedic surgery, piezosurgery

## Abstract

**Background:** The piezoelectric saw is a technology used in osteotomies, providing precise and minimally invasive cuts, especially in areas close to vital structures. Despite its advantages, limitations such as prolonged surgical time and restrictions in use for larger bones have motivated the development of ultrasonic tips with more efficient geometries. **Methods:** A laboratory trial was conducted with 40 ultrasonic tips (*n* = 40), divided into 2 groups: the test group (*n* = 20), with an ultrasonic tip featuring a multidirectional angular cutting-tooth geometry, and the control (*n* = 20), with a straight-tooth ultrasonic tip. Two operators performed standardized osteotomies on synthetic bone blocks, with monitoring of variables including cutting time (in seconds), maximum block and blade temperature (in °C), and bone mass loss (in grams). Sample randomization was block-based, and blade coding ensured operator and evaluator blinding. **Results:** The results showed a statistically significant reduction of approximately 26% in cutting time with the multidirectional ultrasonic tips (Test = 52.85 s; Control = 71.55 s; *p* < 0.001), regardless of the operator. No significant differences were observed between groups regarding maximum bone temperature (Test = 30.45 °C; Control = 29.40 °C; *p* = 0.337), blade temperature variation (Test = 5.30 °C; Control = 4.10 °C; *p* = 0.337), overall temperature variation (Test = −0.19 °C; Control = 0.06 °C; *p* = 0.285), or bone mass loss (Test = 0.1355 g; Control = 0.0350 g; *p* = 0.387). A significant interaction between operator and blade type in some variables, such as bone temperature variation (*p* = 0.001), reinforces the influence of technical experience on the results. **Conclusions:** The multidirectional angular geometry of the ultrasonic tip significantly improves cutting efficiency without compromising thermal safety, representing a promising advancement for optimizing osteotomies in surgical settings. The use of this new geometry may enhance productivity, particularly in complex procedures, and deserves future clinical investigation to expand its applicability across different surgical specialties, including orthopedics.

## 1. Introduction

Osteotomies are part of several surgical specialties within surgery and have different techniques to achieve different objectives when performing bone cutting, such as providing surgical access, correcting deformities, removing autologous bone grafts, debridement, lengthening and bone transport, preparing implants or even removing them, among others [1]. Over time, several technologies have been created to assist and increase the precision of osteotomy procedures, such as the use of osteotomes or chisels, steel cable saws (Gigli saw), drills, oscillating saws, piezoelectric saws and lasers [1,2,3].

In the field of oral and maxillofacial surgery, the use of the piezoelectric saw emerged through the adaptation of a technology present in the dental office to use this device for osteotomy of bones in areas with noble and delicate structures [2,4]. The piezoelectric saw uses ultrasonic technology due to the piezoelectric effect, in which the passage of an electric current through ceramics and crystals causes oscillations in the material and vibration at a frequency of 25–34 KHz, which provides the ability to cut only solid and mineralized materials [2,5,6,7]. This capability offers significant advantages over traditional techniques such as high-speed drills and oscillating saws, as it does not cause damage to soft tissue, as this frequency is specific for cutting bones, nor does it cause an increase in temperature with the possibility of bone necrosis [2,7].

The cutting device consists of a motor, which is coupled to a hose and a device that fits the ultrasonic tip. At the center of the device is the piezoelectric motor, which, when supplied with an alternating current, vibrates at ultrasonic frequencies. These vibrations are transmitted to the blade or ultrasonic tip of the saw, allowing for extremely precise and minimally invasive cuts. Unlike traditional saws, the ultrasonic tip does not rotate or oscillate, but vibrates at low amplitudes and high frequencies, allowing it to cut mineral tissue, such as bone, without damaging adjacent soft tissue. It also has an irrigation system attached to the blade that allows for cooling and less thermal damage to the bone [6,8,9]. This method results in less tissue trauma, lower risk of injury, and faster postoperative recovery for the patient [2,5,7].

Cutting precision, reduced trauma to adjacent tissues, and improved operational visibility are all aspects highlighted in several studies. For example, studies show that the piezoelectric saw not only better preserves bone tissue integrity but also minimizes the risk of thermal and mechanical damage, which is crucial for postoperative recovery, bone healing, and patient rehabilitation [10,11,12,13].

Comparative studies between piezosurgery and high-speed rotary instruments indicate that, although piezosurgery can prolong surgical time due to its gentler cutting technique, it tends to be safer for less experienced surgeons, protecting delicate anatomical structures and reducing the incidence of postoperative complications [6,14,15]. Despite the piezoelectric saw’s numerous advantages, particularly in terms of precision and safety, this technology has some disadvantages that may influence the choice of surgical method in certain situations. One of the main disadvantages is the prolonged operative time. Studies indicate that procedures performed with the piezoelectric saw can be significantly longer when compared to traditional methods using high-speed drills or oscillating saws [4,6,7,10]. Furthermore, the learning curve for efficient use of piezoelectric technology can be challenging for less experienced surgeons, requiring specific training and constant practice to achieve the dexterity necessary to operate the machine effectively [7,8,10].

Conventional straight-tooth piezoelectric blades may experience progressive mechanical resistance during osteotomy, which can increase cutting time, promote blade wear, and impair debris evacuation—phenomena already well-described in other bone-cutting instruments such as oscillating saws. Recent studies on oscillating saw mechanics have demonstrated that linear blade trajectories can elevate cutting forces, lead to ploughing, and increase the risk of structural fatigue and breakage, in addition to promoting the accumulation of bone debris that further reduces efficiency [11,12,13]. These biomechanical challenges are analogous to those encountered during ultrasonic osteotomies, supporting the rationale for exploring alternative blade geometries—such as multidirectional angular-tooth designs—aimed at improving efficiency and thermal safety.

**Figure 1:**
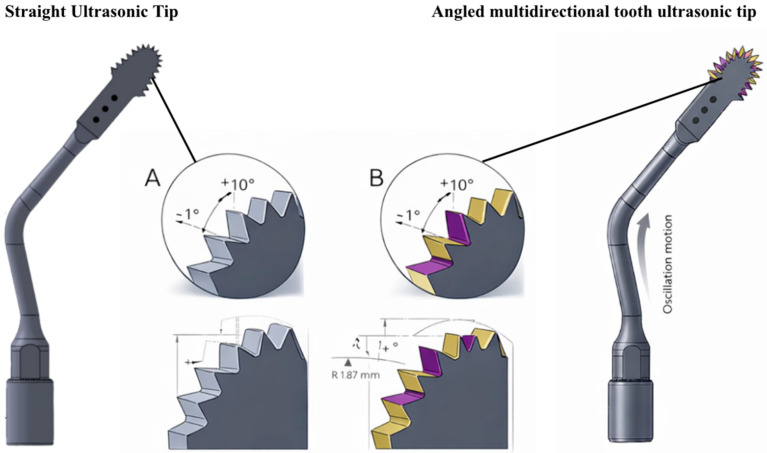
Schematic comparison of the ultrasonic cutting tips evaluated in this study. The straight ultrasonic tip (**left**) presents a conventional uniform tooth geometry, while the angled multidirectional tooth ultrasonic tip (**right**) exhibits an alternating angular tooth design. Magnified views (**A**,**B**) illustrate the tooth geometry, highlighting the rake angle (+10°), clearance angle (−1°), and the alternating angular configuration (±10°) of the cutting edges. The diagrams also depict the cutting-edge radius (R = 1.87 mm) and the oscillatory motion of the ultrasonic device. These original schematic illustrations were created specifically for the present study to demonstrate the geometric differences between the two tip designs. (Source: Author 2024).

The hypothesis raised in this study suggests that changes in the geometry of the blade teeth can significantly influence the bone cutting process. Existing ultrasonic tips have a linear dentition (Figure 1A) and can induce additional stress that is not compensated by ultrasonic microvibration, resulting in reduced cutting efficiency. This phenomenon can prolong the procedure, increase the risk of tip breakage due to high mechanical loads, and complicate the osteotomy process due to the accumulation of bone debris in the blade channels, which obstructs the cutting area as the blade penetrates deeper into the bone.

To address these limitations, a new geometry for ultrasonic tips was developed, characterized by an angular and multidirectional toothing, alternating right- and left-hand angles along the active part of the tip (Figure 1). The hypothesis of this study is that the introduction of an angular multidirectional toothing geometry in the ultrasonic tips can reduce the effort required for cutting, heat generation and procedure time, improving the overall effectiveness of the piezoelectric technique in bone osteotomies. The aim of this study was to compare the performance of ultrasonic tips with straight teeth with multidirectional angled teeth in terms of cutting efficiency, measuring variables such as osteotomy time, blade and bone block temperature, in addition to the analysis of bone residues (bone mass loss) in an experimental study.

## 2. Methods

The present study was a laboratory trial. In this study, the methodology employed involved the use of synthetic bone blocks for biomechanical testing to simulate bone conditions in a controlled environment to test two types of ultrasonic cutting saws (piezoelectric saws) with different dentition designs. A total of 40 ultrasonic tips samples (*n* = 40) were used, divided equally between the test group (multidirectional angle ultrasonic tips—*n* = 20) and the control group (straight dentition ultrasonic tips—*n* = 20) (Figure 1). Each sample was meticulously prepared and subjected to cutting procedures to evaluate variables such as cutting time, bone and blade temperature after cutting, and bone mass loss of the block.

To increase this study’s internal validity and minimize potential bias, the surgeons responsible for the cutting and the data collector were blinded to the saws and groups. To this end, the ultrasonic tips were coded so as to not reveal to the operator whether they were the blade with angular multidirectional geometry or the blade with straight teeth. Sample preparation and blade coding were performed by a third researcher, who was not involved in data collection, ensuring that the surgeons used the blades without prior knowledge of the group to which they belonged, since the type of ultrasonic tip cannot be identified by eye. Furthermore, the data collector, responsible for recording cutting time, bone temperature, and bone residue analysis, was also blinded to the experimental groups, avoiding any influence on the collection and interpretation of the results.

The bone blocks were cut using ultrasonic tips of the same shape, the only difference being the multidirectional cutting geometry (Crossonic—ByPro, São Paulo, Brazil) in the experimental group and straight-toothed tips (Straight Tip—Bypro, São Paulo, Brazil) in the control group, supplied by the same manufacturer. Thermocouples were strategically positioned 5 mm away from the saw cut to measure temperatures during cutting and assess the thermal impact on the bone. Furthermore, cutting times were timed to the nearest second to analyze blade efficiency. Bone residue analysis was performed by weighing the samples before and after cutting with a precision balance (Sartorius BP 210 S, Goetemborg, Sweden) to quantify material loss. The methodology will be detailed below.

**Figure 2:**
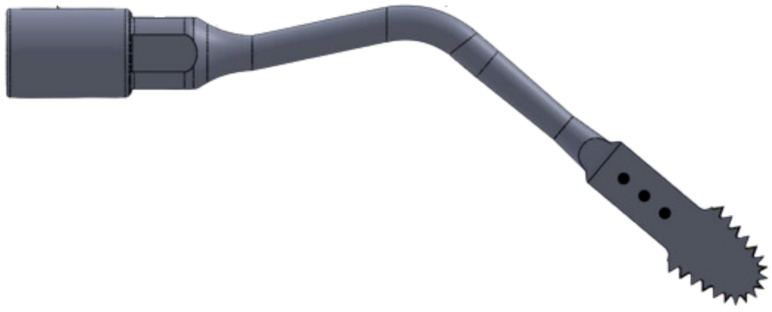
Piezoelectric Saw—13 mm active length, with a width ranging from 3.3 mm to a thickness of 0.35 mm. (Source: Author 2024).

Experimental groups: the samples were divided into two groups according to Figure 2:

- Group 1 Test: use of ultrasonic tip with angular multidirectional cutting geometry (*n* = 20).
- Group 2 Control: use of straight dentition ultrasonic tips (*n* = 20).

This study is an experimental laboratory investigation conducted exclusively on synthetic bone models, without the involvement of human participants, animals, or patient data. Therefore, it does not require ethical approval, informed consent, or adherence to clinical research standards such as the Declaration of Helsinki. The research protocol was formally reviewed by the internal postgraduate academic review committee (AVAP) under protocol number 3065, this study was approved and classified as not requiring submission to an institutional ethics committee. Additionally, this study was not a clinical trial and received external funding from the manufacturer who provided all the materials and ultrasonic tips for this study. Accordingly, the following statements apply: “Human Ethics and Consent to Participate Declarations: not applicable” and “Clinical trial number: not applicable.”

The biomechanical test block called “30 PCF Test Body with 3 mm Bicortical,” code 12214, offered by Nacional Ossos Inc^®^ (Jaú, São Paulo, Brazil), was used to perform the osteotomies with the saws. This block is ideal for simulations in controlled environments due to its bicortical composition, which includes a 3 mm upper and lower layer with a density of 40 PCF (0.96 g/cm^3^), mimicking cortical bone, and a core with a density of 30 PCF (0.16 g/cm^3^), which simulates cancellous bone. This block is specifically designed to evaluate the anchorage of joint prostheses, implants, and other biomaterials, making it a choice for evaluating accuracy and biomechanical impacts under simulated clinical testing conditions. A total of 40 synthetic test bone samples of the same size were used (5 × 5 × 3 cm) with a 3 mm hole to measure the temperature of the cut inside the bone with the thermocouple.

**Figure 3:**
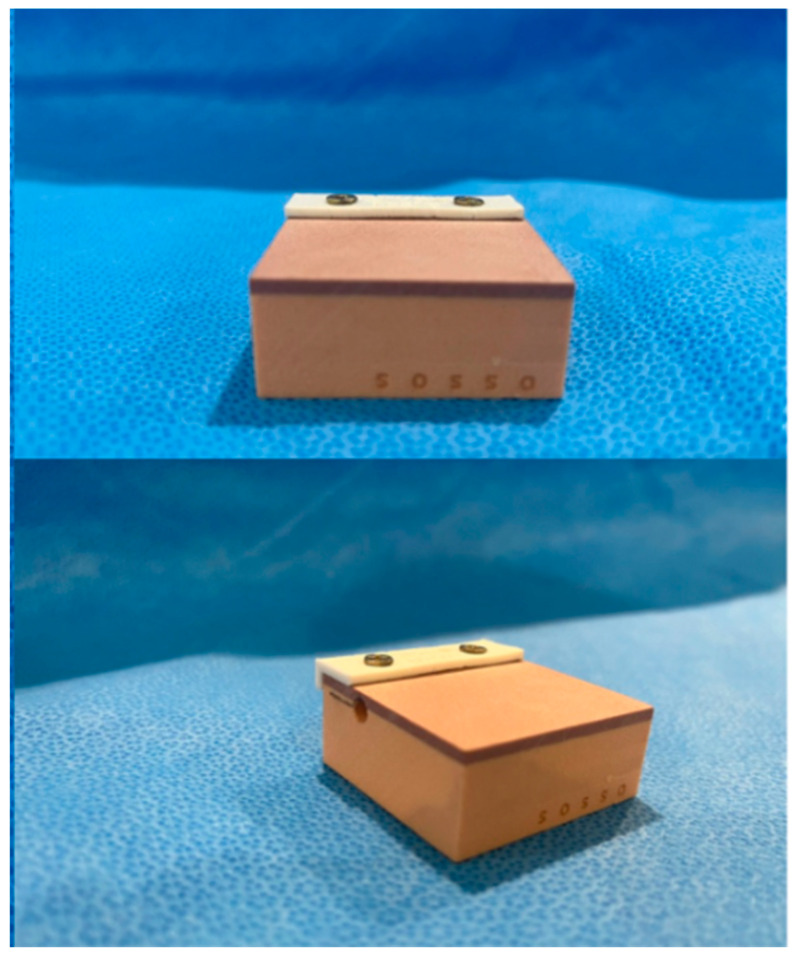
Image of the bone blocks with the fixative cutting guide on top (white part).

**Figure 4:**
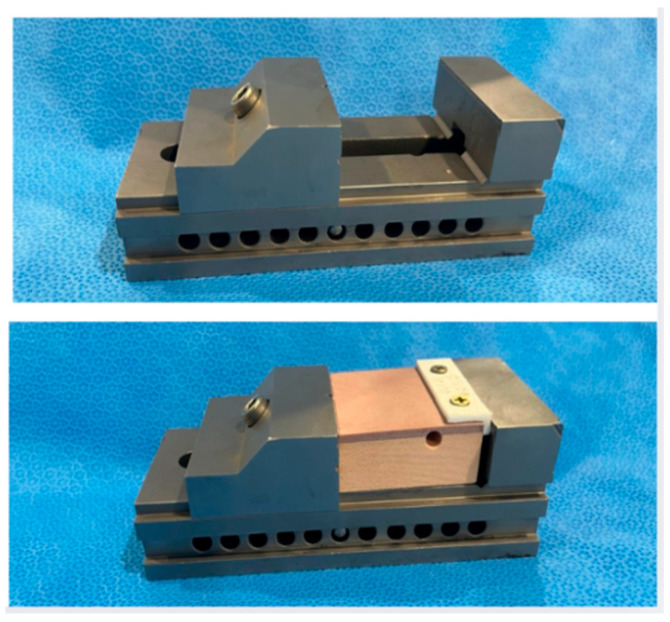
Image of the bone block fixation instrument on the table.

To ensure standardization of cutting direction and distance, a specific cutting guide measuring 1 × 5 cm was developed on a 3D printer and attached to each bone block with two screws (Figure 3 and Figure 4). This ensured consistent cutting direction and timing, as it is operator dependent.

The sample size was determined based on a pilot study with 10 samples in each group (*n* = 20). The preliminary results allowed us to estimate data variability and calculate statistical power (power test), adopting a significance level of 5% (α = 0.05) and a minimum power of 80% (1 − β = 0.80). Based on these parameters, 40 blades were used in the main study, with 20 for the control group and 20 for the test group. This number was considered adequate to reduce the probability of Type II error and ensure sufficient robustness for detecting relevant differences between the groups. Furthermore, this sample size, larger than that used in previous studies, increases sensitivity for identifying even subtle differences in cutting speed and heat generation between multidirectional piezoelectric blades and conventional ones. This strategy strengthens the reliability of the results, improves external validity and reproducibility, and ensures that the conclusions faithfully reflect the inherent characteristics of the slides tested.

The motor used in this study is the Mectron^®^ Piezosurgery Touch model (Mectron^®^, Carasco, Italy), operating in a frequency range of 24–34 kHz, in cortical bone cutting mode. The blade cooling system was used with 0.9% saline solution in this study at intensity number 1 (range 1–5).

**Figure 5:**
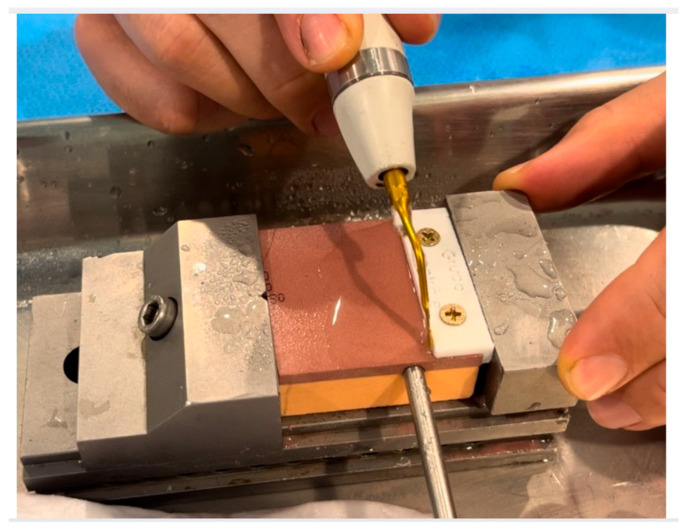
Image of the study procedure: Bone block with thermocouple and saw performing a straight longitudinal cut up to its maximum active length of 13 mm along the entire length of the bone block. Source: author 2024.

The synthetic bone block samples, along with their cutting guides, were fixed to stable supports to ensure precision during the cuts. A simple osteotomy was performed until the saw cutting area was completely inserted into the entire cortical and cancellous region, following the cutting guide. These osteotomies were performed by two surgeons previously trained with the blade, and a cutting guide was used to facilitate guidance and standardize the entire osteotomy path. Furthermore, the same operators performed the same number of osteotomies and ultrasonic tips (10 in the control group and 10 in the test group—*n* = 20 for each operator) to avoid inter-operator bias. Both operators and evaluators were blinded (Figure 5).

Temperature was recorded using a type-K thermocouple positioned in a fixed and reproducible 3D coordinate relative to the osteotomy. For all samples, the thermocouple tip was placed 5 mm lateral to the cutting line, measured perpendicularly from the cortical surface, and aligned with the midpoint (50%) of the standardized 20 mm osteotomy path, ensuring uniform longitudinal positioning. A 1.2 mm pilot canal was drilled perpendicular to the cortex to a depth of 3 mm, allowing for consistent insertion of the thermocouple at the cancellous–cortical interface.

The probe was stabilized to prevent displacement during irrigation and vibration. All measurements were performed using the same positioning jig, guaranteeing identical geometric placement in all 40 samples and ensuring reliability and reproducibility of the thermal data.

The duration of each cut was measured with timers that began counting from the initial contact of the saw with the bone until the block was completely cut to the saw’s cutting thickness. The saw temperature before and after the cut was measured with a digital laser thermometer. The osteotomies were performed in the laboratory in the same location at an ambient temperature of 21 °C to standardize possible changes related to the environment.

### 2.1. Independent Variable

This study used ultrasonic tips with the same formats: 13 mm long, 3.3 mm wide, and 0.35 mm thick. The difference between the two groups is the geometry of each saw’s teeth. These saws are manufactured and supplied by BYPRO Medical do Brasil ^®^ (Cajamar, São Paulo, Brazil) (Figure 1 and Figure 2). All BYPRO Medical products^®^ are certified by ANVISA and are already used clinically in osteotomy procedures.

#### 2.1.1. Straight Teeth Ultrasonic Tips Control

These tips feature a blade configuration known as a microsaw, with a dimensional composition of 13 mm active length, with a width ranging from 3.3 mm to a thickness of 0.35 mm. The straight-tooth ultrasonic tip presents a uniform linear dentition with constant rake and clearance angles along the cutting edge. In contrast, the multidirectional-angled ultrasonic tip incorporates an alternating angular tooth geometry, designed to modify cutting directionality and efficiency (Figure 1A).

#### 2.1.2. Ultrasonic Tip with Angular Multidirectional Cutting Geometry

**Figure 6:**
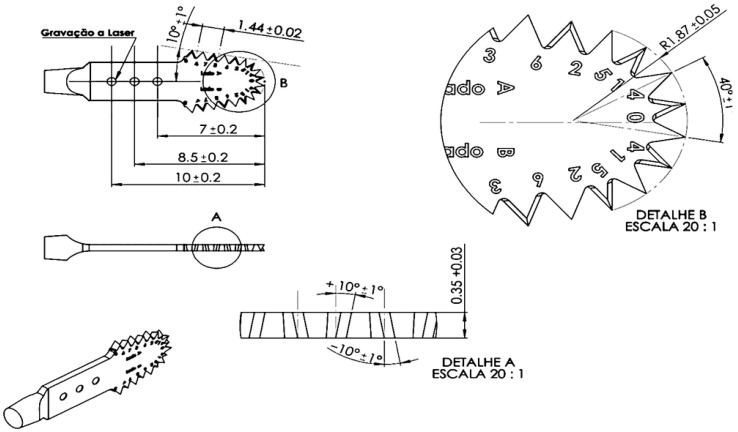
Technical schematic of the multidirectional-angled ultrasonic tip. Detailed representation of the tooth geometry, showing the alternating angular design (±10°), rake angle (10°), clearance angle (1°), tooth pitch, cutting edge radius (R = 1.87 mm), and blade thickness (0.35 mm). Tolerances for each parameter are indicated. Details A and B highlight the angular transition zones and dentition profile at 20:1 scale. Source: author 2024.

This tip has the same shape as the control group and the only difference is the angular multidirectional dentition geometry, alternating and intercalating the angulation of the teeth to the right and to the left along the active part of the tip (Figure 1B). According to the manufacturer’s technical specifications, the multidirectional tip features an angular alternation of ±10° (±1° tolerance) between adjacent cutting segments, with a primary tooth angle of 40° (±1°). The rake angle is 10° (±1°), while the clearance angle is 1° (±1°). Tooth pitch and spacing are standardized, with measured distances of 7.0 ± 0.2 mm, 8.5 ± 0.2 mm, and 10.0 ± 0.2 mm between reference points along the cutting edge. The tooth height is 1.44 ± 0.02 mm, and the cutting-edge radius at the transition zone is R = 1.87 ± 0.05 mm (Figure 6).

### 2.2. Main Outcome Variable(s)

#### 2.2.1. Cutting Time (Seconds)

The procedure for each sample included initial preparation of the equipment and secure attachment of the sample to the cutting site and the use of a guide attached to the part to ensure accuracy and standardization of the cut. Cutting time was meticulously timed from the start of the cut to the complete insertion of the blade into its cutting area across the designated section of the sample. This measurement was taken using a digital stopwatch to ensure accurate capture of the cutting duration in seconds by an independent evaluator. The times recorded for each cut in both groups were compiled and statistically analyzed to determine whether the new tip geometry offers a significant advantage in cutting speed compared to traditional tips.

#### 2.2.2. Bone Temperature (°C)

During the cutting tests in this study, bone temperature was monitored using thermocouples inserted into a 3 mm hole in the bone blocks, which were strategically positioned 5 mm from the cutting area within the bone. The initial temperature, maximum temperature, and temperature at the end of the cut were recorded and recorded in degrees Celsius (°C) in a table for each sample.

#### 2.2.3. Blade Temperature (°C)

A laser thermometer (GM 400—GAMA^®^, São Paulo, SP, Brazil) was used to measure and record the temperature generated in the blade’s cutting area immediately before and after each cutting procedure performed with the ultrasonic tips. This approach allows for accurate, non-contact assessments, ensuring that measurements are made safely and non-intrusively, without altering the condition of the equipment or the material being tested. The blade temperature was collected before cutting and immediately after the osteotomy was completed.

#### 2.2.4. Residue Analysis and Weighing (g)

The precision balance (Sartorius BP 60 S^®^, Göttingen, Germany) was used to weigh the samples and calculate the bone loss generated during cutting tests with the ultrasonic tips. Each sample was weighed before and after the cutting procedures to determine the exact amount of material removed. To ensure accurate bone loss analysis, the blocks were weighed after two days of the experiment to dry, since the cuts were made with saline solution refrigeration, changing the weight of the block immediately after the experiment with the solution.

### 2.3. Data Analysis and Statistical Methodology

#### 2.3.1. Defining Variables

Variables related to the cutting performance and thermal behavior of the piezoelectric blades were analyzed. Cutting time (seconds) was defined as the interval required to complete each osteotomy until the blade was fully inserted into the entire length of the bone block. The bone temperature before cutting (°C) corresponded to the initial temperature of the block before incision, while the bone temperature after cutting (°C) represented the maximum temperature reached immediately after the end of the cut. The blade temperature before cutting (°C) indicated the initial blade temperature, and the blade temperature after cutting (°C) corresponded to the maximum temperature recorded immediately after the end of the osteotomy. Bone mass before cutting (g) was measured on a precision scale to three decimal places, reflecting the initial weight of the block before the procedure, while bone mass loss (g) represented the difference in weight between the block before and after cutting, also expressed to three decimal places. Finally, bone mass loss (%) was calculated as the percentage ratio of bone removed in relation to the initial mass, providing an additional measure of the efficiency and precision of the cutting performed.

#### 2.3.2. Statistical Methodology Used

The data were summarized by summary statistics, such as mean, standard deviation, median, minimum and maximum value found in the sample.

All parameters were tested for normality (Shapiro–Wilk test), and the results showed significant deviations from the normal distribution. Therefore, two statistical analyses were performed: one ignoring the operator and the other using the difference between the two operators. The groups (test and control) were compared using a fixed-factor analysis of variance (ANOVA) model but applied to data transformed into ranks. For better visualization of the study data summary, boxplot graphs were constructed according to the type of probe used.

For each operator analysis, the groups (test and control) and operators were compared using a two-factor analysis of variance (ANOVA) model but applied to data transformed into stations. To better visualize the ANOVA results, line graphs were constructed to visualize the comparison between tip types and between operators.

The software Minitab (version 19.1, State College, PA, USA) was used to create tables and graphs, as well as all analyses used in this work, considering a significance level for *p* values ≤ 0.05.

## 3. Results

### 3.1. Cutting Time

Operator’s 1 data indicated that the use of the test tip resulted in a shorter cutting time (M = 55.50 s; SD = 7.86) compared to the control tip (M = 80.80 s; SD = 19.34). Similarly, for Operator 2, the cutting time was shorter with the test tip (M = 50.20 s; SD = 7.21) compared to the control tip (M = 62.30 s; SD = 13.60).

The analysis of variance based on the ranks showed a statistically significant effect for both the factor *tip type* (F = 26.56; *p* < 0.001), as for the factor *operator* (F = 8.41; *p* = 0.006), indicating that the cutting time was significantly shorter with the test tip, regardless of the operator, and that operator 2 performed better in terms of time. The interaction between operator and tip type, however, was not statistically significant (F = 1.67; *p* = 0.204), suggesting that the effect of tip type on cutting time was similar among operators.

When the data were analyzed in aggregate, ignoring the operator factor, it was observed that the test group presented a significantly shorter mean cutting time (M = 52.85 s; SD = 7.83) compared to the control group (M = 71.55 s; SD = 18.84), with a statistically significant difference (F = 21.90; *p* < 0.001). The graph reinforces this evidence, demonstrating less variability in the test group and highlighting the greater dispersion and presence of outliers in the control group (Table 1 and Table 2).

### 3.2. Bone Temperature During Cutting (Maximum)

The analysis of the maximum bone temperature during cutting, considering the operators separately, revealed variations between the test and control groups. For operator 1, the maximum temperature was similar between the tip types, with a mean of 28.9 °C (SD = 1.79) for the test tip and 29.2 °C (SD = 1.14) for the control tip. For operator 2, a higher mean was observed with the test tip (32.0 °C; SD = 4.69) compared to the control tip (29.6 °C; SD = 3.44).

The analysis of variance for continuous variables did not identify statistically significant differences between the tip types used (F = 0.14; *p* = 0.711), nor between the operators (F = 0.44; *p* = 0.510). The interaction between tip type and operator was also not significant (F = 2.63; *p* = 0.114), indicating that the variation observed between the groups can be attributed to random factors and not to a systematic effect of the variables studied.

**Figure 7:**
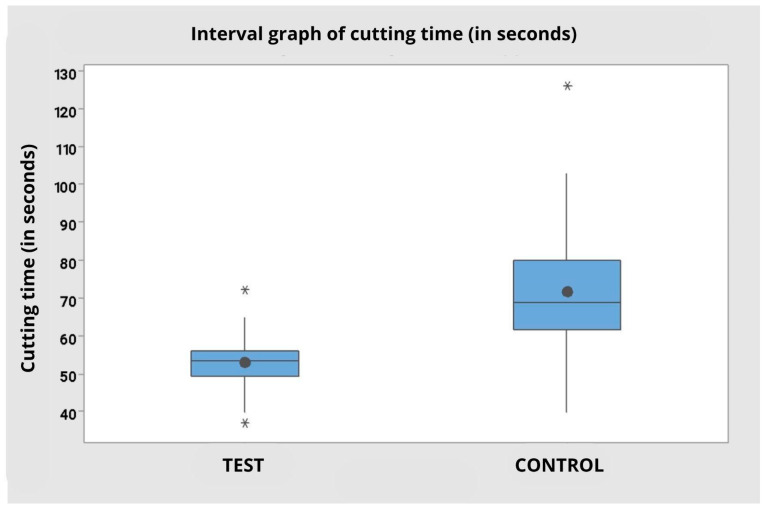
Interval graph of cutting time (in seconds) comparing the Control and Test groups, regardless of the operator. Note a shorter cutting time with less variation in the test group compared to the control. Presence of outliers represented by * in both groups.

When considering the grouped data, disregarding the operator factor, the test group presented a mean bone temperature of 30.45 °C (SD = 3.80), while the control group presented a mean of 29.40 °C (SD = 2.50). The analysis of variance by ranks reinforced the absence of statistical significance between the groups (F = 0.95; *p* = 0.337), corroborating that there was no relevant difference between the tip types with regard to maximum bone temperature (Figure 7).

The boxplot illustrates the overlap of interquartile ranges and the presence of outliers in both groups, with a slight tendency for greater variability in the test group.

Table 3, Descriptive statistics of maximum bone temperature (in degrees Celsius) comparing the control group with the test for Operator 1, 2 and pooled data.

### 3.3. Bone Temperature Difference (Final—Initial)

The evaluation of the difference between the final and initial bone temperature during cutting indicated relevant variations between operators and tip types (Table 4). For operator 1, the mean temperature difference was 3.8 °C (SD = 1.69) for the test tip and 4.8 °C (SD = 1.03) for the control tip. For operator 2, the test tip resulted in a significantly greater difference (M = 6.80 °C; SD = 4.13) than the control tip (M = 3.40 °C; SD = 3.95).

The analysis of variance by ranks showed that there was no statistically significant effect for the tip type alone (F = 1.21; *p* = 0.279) nor for the operator (F = 0.55; *p* = 0.463). However, the interaction between operator and tip type was statistically significant (F = 11.89; *p* = 0.001), indicating that the effect of the tip type on the temperature difference depends on the operator.

**Figure 8:**
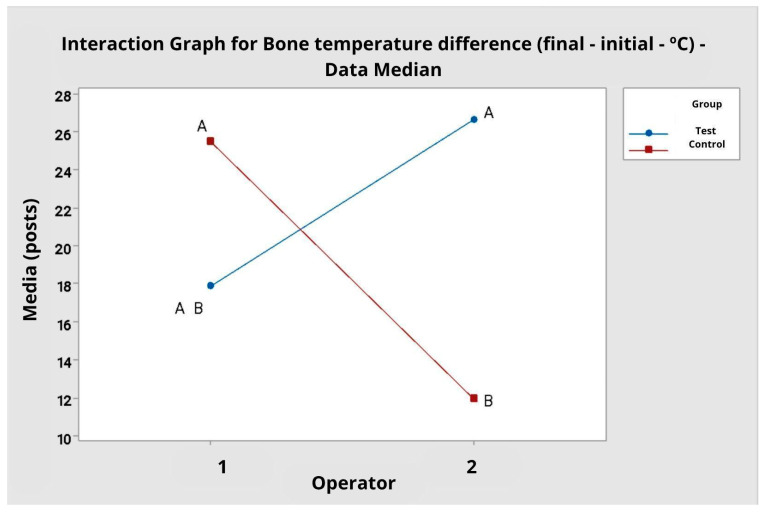
Interaction plot for bone temperature difference. Data averaged by operator. A and B: clustering information, according to Tukey’s multiple comparisons.

**Figure 9:**
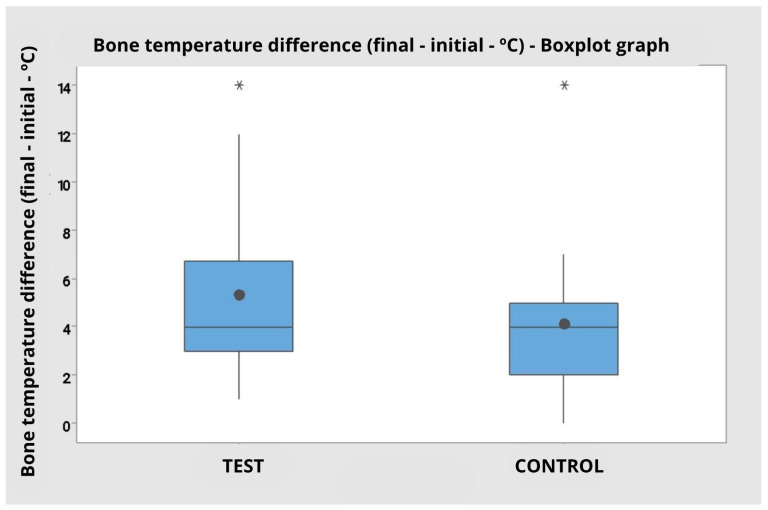
Boxplot graph for bone temperature difference comparing the test and control groups regardless of the operator. Presence of outliers represented by * in both groups.

**Figure 10:**
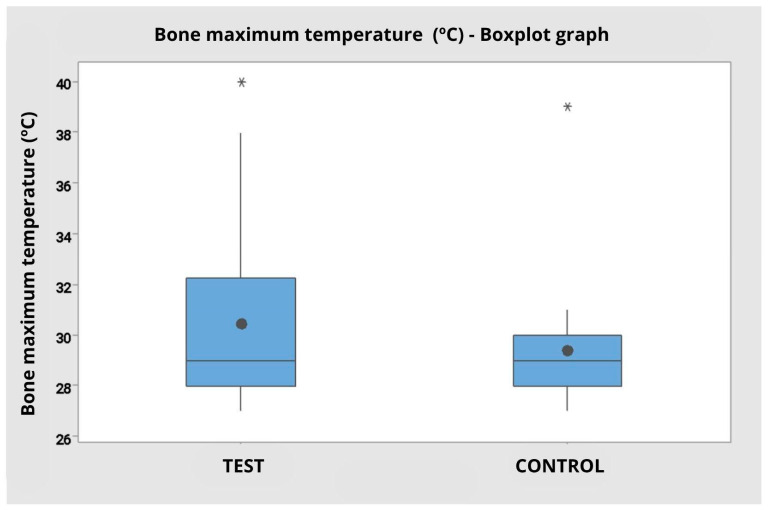
Graph of maximum bone temperature (in degrees Celsius) comparing the Control and Test groups independent of the operator. Presence of outliers represented by * in both groups.

Tukey’s multiple comparisons confirmed this interaction. For operator 1, there was no significant difference between the test and control tips. Conversely, for operator 2, the test tip resulted in a significantly greater bone temperature elevation (mean difference of 6.80 °C) compared to the control tip (mean difference of 3.40 °C), with distinct clusters (A vs. B). This is corroborated by the interaction plot, which shows crossed lines between groups and operators, and by the boxplot (Figure 8 and Figure 9), which indicates greater dispersion in the test group’s values (Figure 10).

When the data were grouped, disregarding the operator factor, the mean temperature difference was 5.30 °C (SD = 3.43) for the test tip and 4.10 °C (SD = 2.90) for the control tip. Analysis of variance by ranks, however, did not indicate a statistically significant difference between the groups (F = 0.95; *p* = 0.337).

### 3.4. Blade Temperature Before and After Cutting (°C)

The evaluation of the blade temperature variation during cutting (difference between final and initial temperature) showed mean values close to zero for most groups. For operator 1, the temperature difference was similar between the groups: −0.35 °C (SD = 0.77) with the test tip and −0.38 °C (SD = 0.82) with the control tip. For operator 2, the mean difference was −0.03 °C (SD = 0.90) with the test tip and 0.50 °C (SD = 0.67) with the control tip.

Analysis of variance by rank revealed a statistically significant difference between operators (F = 5.70; *p* = 0.022), with operator 2 presenting a greater increase in blade temperature during the procedure, regardless of the type of tip used. However, no significant differences were observed between tip types (F = 1.34; *p* = 0.255), nor was there any interaction between operator and tip type (F = 1.51; *p* = 0.227).

**Figure 11:**
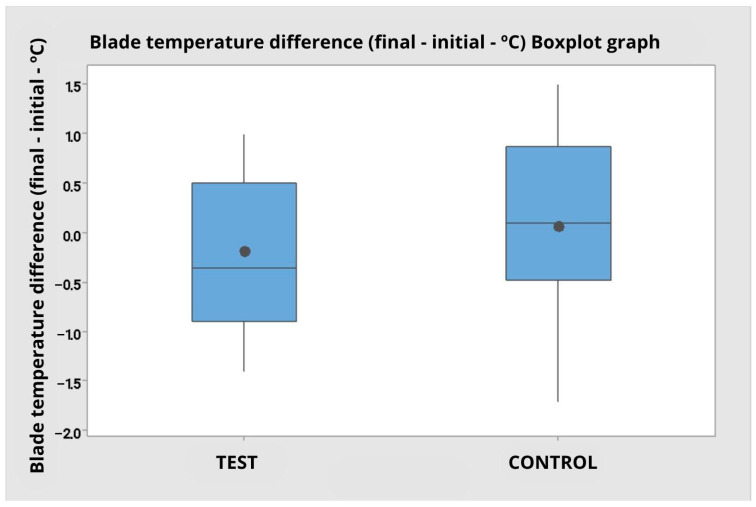
Boxplot graph for blade temperature difference (final—initial) comparing the test and control group independent of the operator.

The global analysis, pooling the data from both operators, reinforced the absence of statistical difference between the tip types (M test = −0.19 °C; M control = 0.06 °C; F = 1.18; *p* = 0.285). The boxplot (Figure 11) shows that both groups have a median close to zero, with a symmetrical distribution and similar amplitude, with no clear trend of increasing or decreasing temperature associated with the tip type.

Table 5, Descriptive statistics of the blade temperature difference (final—Initial) (in degrees Celsius) comparing the control group with the test for Operator 1, 2 and grouped data.

### 3.5. Mass Loss (g)

The variable mass loss during the procedure presented different means between operators and tip types, but without statistical significance. For operator 1, mass loss was higher in the test group (M = 0.239 g; SD = 0.433), with wide variability and the presence of outliers, compared to the control group (M = 0.0410 g; SD = 0.0420). For operator 2, the values were similar between the groups, with means of 0.0320 g (SD = 0.0210) for the test tip and 0.0290 g (SD = 0.0099) for the control tip.

Analysis of variance by ranks indicated no statistically significant difference between tip types (F = 0.76; *p* = 0.390), between operators (F = 0.46; *p* = 0.501), and in the interaction between operator and tip type (F = 1.18; *p* = 0.284).

**Figure 12:**
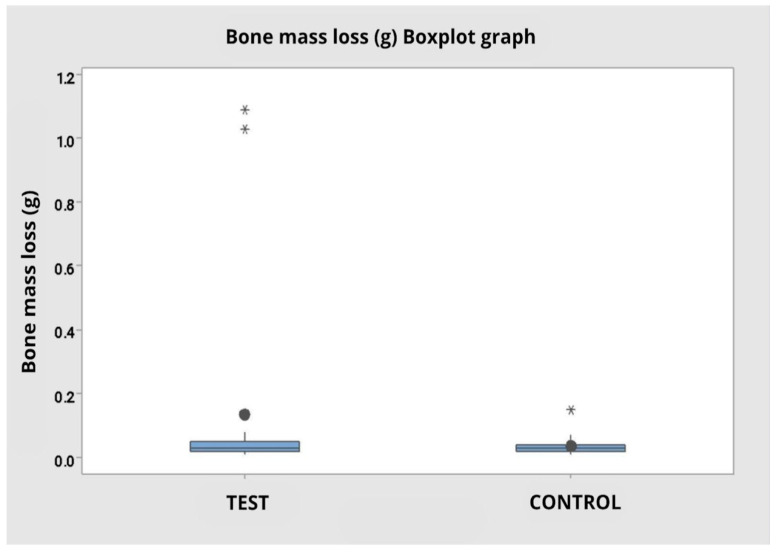
Boxplot graph for bone mass loss comparing the test and control groups regardless of the operator. Presence of outliers represented by * in both groups.

In the grouped analysis, disregarding the operator factor, the mean mass loss was greater in the test group (M = 0.1355 g; SD = 0.3168) compared to the control (M = 0.0350 g; SD = 0.0304); however, it did not present a statistically significant difference (F = 0.76; *p* = 0.387) (Figure 12).

## 4. Discussion

Piezoelectric blades have been used in oral and maxillofacial surgery for over 30 years, with different manufacturers and formats available. However, until now, there had been no descriptions in the literature of tooth geometry modifications similar to those proposed in this study [2,16]. The results demonstrated that the multidirectional toothed ultrasonic tip significantly reduced cutting time compared to the straight toothed ultrasonic tip, with an average difference of 26%, confirming the initial hypothesis that the new design could increase the efficiency of the piezoelectric technique. This gain is particularly relevant because prolonged operative time is considered the main limitation of piezosurgery.

Previous studies have highlighted this limitation, especially in procedures requiring precision, such as craniomaxillofacial osteotomies, craniotomies, or orthopedic surgeries for hallux valgus correction [17,18,19]. Gleizal et al. conducted a study comparing procedure times for craniotomies, which demonstrated a mean time of 1 h and 10 min using a piezoelectric saw compared with a mean time of 43 min using a craniotome, representing an approximately 60% increase in the time for craniotomies performed with the piezoelectric saw compared with the conventional craniotome [6]. Russo et al. reported in their clinical study for hallux valgus correction that osteotomy times were 3 min using an oscillating saw and 10 min using a piezoelectric saw [3]. These data reinforce that, historically, time has been a barrier to widespread adoption of the technology. In this context, the 26% reduction in the present study brings the efficiency of piezosurgery closer to the performance of conventional methods, representing an important advance.

From a biomechanical standpoint, the angular alternation of the teeth in the multidirectional blade reduces the area of effective contact with the bone, which reduces mechanical cutting resistance. Furthermore, the multidirectional design of the teeth facilitates the continuous removal of bone debris, preventing obstructions that often prolong the procedure time with straight-tooth blades. These factors explain why cutting becomes faster without a proportional increase in heat generation or bone loss.

The multidirectional angular geometry of the ultrasonic tip introduces localized changes in cutting edge orientation that may theoretically increase stress concentration at angular transition zones, potentially influencing fatigue behavior and microfracture risk over repeated use. Although no macroscopic tip failure was observed during the experimental protocol, this study was not designed to evaluate long-term durability, wear patterns, or fracture resistance. In clinical practice, wear or microdamage may be indirectly monitored through visual inspection, changes in cutting efficiency, or increased operative time. Dedicated mechanical durability testing, including cyclic fatigue analysis and finite element modeling, would be required to quantify stress distribution and lifespan under repeated loading conditions. These aspects represent important directions for future investigations and are acknowledged as limitations of the present in vitro study.

Our findings align with recent evidence from oscillating bone sawing research, which demonstrates that tool geometry plays a fundamental role in cutting efficiency, thermal generation, and chip evacuation. Wang et al. (2025) showed that conventional blades with large negative rake angles experience excessive ploughing forces, inefficient chip evacuation, and accelerated tip wear—factors that significantly elevate cutting temperature and compromise surface integrity [11]. Their work demonstrated that geometric optimization capable of reducing ploughing and enhancing chip fragmentation markedly decreases cutting forces (up to 56%), suppresses peak temperature to 43.3 °C, and limits wall cracking. Although their study focused on a novel flexible-structured oscillating saw, the underlying biomechanical mechanisms are directly comparable to ultrasonic osteotomies: both systems face limitations related to linear cutting trajectories, debris accumulation, and force concentration along the cutting edge. Therefore, the improved performance observed with our multidirectional angular-tooth piezoelectric blade is consistent with the advantages described by Wang et al. (2025), supporting the concept that modifying blade geometry to favor effective chip evacuation and reduce ploughing-induced resistance enhances cutting efficiency and thermal safety across different bone-cutting modalities [12].

Another recurring criticism of the piezoelectric saw is its lengthy learning curve, with greater technical complexity at the beginning of use, as pointed out by several authors [2,6,20,21]. Using a piezoelectric saw requires specific training, as its handling technique differs significantly from conventional instruments. The operator should apply as little pressure as possible during the cut, allowing the ultrasonic microvibrations to act effectively on the bone tissue. This concept may seem counterintuitive, since in traditional cutting methods, it is common to increase the pressure on the blade to speed up the process. However, with the piezoelectric saw, increasing pressure compromises the amplitude of the microvibrations, reducing their cutting effectiveness. Therefore, for optimal performance, smooth, controlled movements with low pressure should be prioritized [20,21]. Gleizal et al. demonstrated that, after two years of continuous practice, there was an approximately 20% reduction in surgical time with the use of ultrasonic technology [6].

Although the learning curve was not formally assessed using novice operators or predefined training metrics, the present findings indirectly highlight the influence of operator experience on piezosurgical performance. In the comparative analysis, operator 2 consistently demonstrated shorter cutting times regardless of the ultrasonic tip used, suggesting greater familiarity with the equipment and improved control of application pressure (*p* = 0.006). A significant operator–tip interaction was observed for bone temperature (*p* = 0.001), indicating that thermal behavior depended on both the tip design and the operator’s handling. While no significant temperature differences were detected for operator 1, operator 2 exhibited higher bone temperature values with the multidirectional tip, potentially reflecting greater applied force or reduced irrigation efficiency during more aggressive cutting. Additionally, a trend toward increased bone mass loss was observed in samples from operator 1, which may indicate lower precision and stability during osteotomy. Together, these findings underscore the relevance of technical proficiency in ultrasonic osteotomy and suggest that multidirectional blade geometry may help reduce osteotomy time independently of operator experience [22]. This characteristic could potentially optimize the learning curve, reduce surgeon fatigue, and improve procedural safety; however, these assumptions remain speculative and warrant formal investigation in future studies specifically designed to evaluate learning effects in less experienced surgeons.

The results of this study demonstrated no statistically significant differences between the groups regarding maximum bone temperature or blade temperature variation during cutting, regardless of the tip type used. In both cases, the average temperatures remained well below the critical limit of 47 °C, recognized in the literature as the point at which thermal necrosis occurs [10]. These findings suggest that both the straight and multidirectional tips exhibit safe thermal behavior. Although statistical superiority of the multidirectional geometry in thermal control was not observed, its design—by reducing friction and potentially improving heat dissipation—may still offer relevant clinical benefits, especially in osteotomies that require precision and tissue conservation. Maintaining temperatures within a safe range is essential to preserve cell viability, promote bone healing, and minimize thermal damage to critical anatomical structures [21].

Studies in the literature usually compare different osteotomy methods with the piezoelectric saw, demonstrating the ability of piezoelectric saws to maintain cooling and a temperature below 47 °C [8,21,22]. Harder et al. compared three piezoelectric saws from different manufacturers, but with the same characteristics and tooth shape, finding better performance in one of the saws from a single manufacturer and a minimal temperature difference between them [23].

Schultz et al. compared different piezoelectric saws that maintained the same straight tooth shape to perform osteotomies and osteoplasties on swine mandibles heated to 36 °C. The methodology used temperature sensors positioned 3 mm deep and 1 mm away from the work site, while osteotomies were performed with a Piezosurgery 3 saw (Mectron, Verona, Italy) and cooled with Ringer’s solution. The intraosseous temperature variation was significant between the different saws, with maximum peaks above 47 °C for short periods, not exceeding 29 s [24].

Adequate irrigation during the use of ultrasonic devices is essential for maintaining temperature and preventing thermal damage to bone tissue [25]. According to Budd et al., the heat generated by ultrasonic devices occurs through three main mechanisms: friction between the ultrasonic tip and the contact surface, the temperature of the coolant used, and the absorption of acoustic energy by the material [25]. Lack of efficient irrigation can lead to temperature increases greater than 10 °C, exceeding the threshold considered safe. Budd et al. demonstrated that the use of 30 mL/min of coolant was effective in preventing significant temperature increases, while lower rates, such as 15 mL/min, still presented the risk of harmful thermal elevation [25]. These findings highlight the importance of combining constant irrigation and adequate volume with ultrasonic techniques, especially in prolonged procedures, to ensure safety and preserve the integrity of surrounding tissues.

The results of this study may have significant potential clinical implications, demonstrating that the multidirectional blade reduces cutting time compared to the conventional blade. These characteristics can directly impact surgical practice by reducing overall procedure time, which in turn reduces the risk of complications such as infection associated with prolonged exposure and excessive bleeding [2]. In complex and long-term surgeries, the efficiency provided by the new geometry can improve patient safety and increase surgical productivity. The reduction in bone heating also reduces the risk of thermal necrosis, favoring tissue preservation and bone healing, which are fundamental elements for the success of the procedure.

All osteotomy methods have specific advantages and disadvantages. Osteotomy performed with an osteotome, for example, is simple, quick, and economical, but its main limitations include the risk of undesirable fracture at the osteotomy site and the much greater effort required by the surgeon during the procedure, which can lead to injury to surrounding vital structures [1]. On the other hand, techniques such as the Gigli saw, oscillating saw, drill, and chisel (De Bastiani technique), widely used in orthopedics, share the disadvantage of generating high temperatures during cutting, which can lead to osteonecrosis and alteration in the histological and cellular structure of the bone, with impaired tissue regeneration and healing, in addition to the increased risk of injury to adjacent soft tissues [1,26,27,28]. In this context, piezosurgery, with lower heat generation and selectivity for bone mineral tissue, demonstrates a significant advantage in safety and preservation of bone viability, reducing the risk of necrosis compared to these traditional osteotomy methods. Furthermore, it does not affect the histological structure of the bone, thus causing better regeneration, greater consolidation potential and less postoperative pain in these patients [2,3,5,8,20,21,26,29,30].

Although the blade temperature did not show a statistically significant difference between the groups, this result can be explained by factors related to the cutting dynamics and the constant irrigation of the blade during cutting. The multidirectional tooth design may generate a temporary temperature increase due to greater cutting efficiency and energy dissipation. However, the significantly shorter cutting time in the test group may have offset this effect, resulting in similar final temperatures. This suggests that the new geometry offers a balance between efficiency and thermal control, without negatively impacting blade safety during the procedure.

During the analysis of the results, outliers were observed in the bone temperature recordings in both groups. One possible explanation for these discrepant values is the inadvertent contact of the saw with the thermocouple during the osteotomy. This contact may have generated an artificial elevation in the reading, not necessarily reflecting the actual thermal behavior of the bone during cutting. This hypothesis is consistent with the fact that, even in these cases, there was no correlation with a consistent temperature increase in the other blocks, reinforcing the interpretation that these extreme values should be attributed to measurement artifacts. The occurrence of these outliers highlights the importance of careful sensor positioning in laboratory studies and suggests that future work may benefit from the use of multiple thermocouples, robots for cutting, or complementary thermal monitoring methods for greater data reliability.

The lack of statistically significant difference in bone loss between the groups reflects the inherent precision of both piezoelectric blade geometries. This high precision minimizes the amount of material removed, ensuring a controlled and conservative cut.

One of the main limitations of this study is the use of synthetic bone blocks as substitutes for human tissue. Although these models have comparable density and structure to cortical and trabecular bone, they do not fully reproduce the anatomical variability, vascularization, and biological response that occur in vivo. In a real surgical environment, factors such as the presence of periosteum, bleeding, bone elasticity, basal body temperature, and interaction with adjacent soft tissues can significantly alter thermal dissipation and cutting resistance. Therefore, although the laboratory findings indicate thermal safety and greater cutting efficiency, their direct extrapolation to clinical practice should be interpreted with caution.

Another important limitation is the lack of histological or cellular assessment of bone viability after osteotomies. Previous studies have shown that microfractures, alterations in the mineral matrix, and osteoblast viability around the cut area can be determinants of healing success [20,21,23,24]. Although temperature and mass loss parameters provide an indirect estimate of procedural safety, this study did not allow us to determine the true impact of multidirectional geometry on cellular integrity and bone regeneration capacity.

Furthermore, the experiment was conducted in a highly controlled laboratory environment, with standardized irrigation, a constant room temperature (21 °C), and a 3D-printed cutting guide to reduce technical variability. These controls increase reproducibility but distance themselves from clinical reality, where factors such as variable irrigation flow, anatomical differences, body temperature, and inadvertent saw movement directly influence the results. Therefore, the observed gains in cutting time may be smaller or more heterogeneous when applied to complex clinical scenarios.

The osteotomies were performed by two different operators, with group randomization and “blinding” of both the operators and the team responsible for data collection, to evaluate the performance of the multidirectional blade at different levels of technical execution. This approach increases methodological robustness by reducing execution and observation bias, in addition to allowing for greater generalization of the results. The inclusion of two operators allowed for indirect assessment of the learning curve and reproducibility of the technique with different profiles, bringing this study closer to clinical reality, where surgeons’ experience can vary widely.

Another point to consider is the dependence on operator skill. Although randomization and blinding reduce bias, clear differences between the two surgeons highlighted the influence of the learning curve. This study did not objectively measure the pressure applied to the blade, which may have influenced both cutting time and thermal variation. The lack of this control makes it difficult to determine whether some of the observed differences were in fact attributed to blade geometry or individual technique.

In the comparative analysis between the two operators, it was observed that the cutting time was consistently shorter for operator 2, which may reflect greater familiarity with the technique and better control of the ideal application pressure (*p* = 0.006). Regarding bone temperature during cutting, there was a significant interaction (*p* = 0.001), indicating that the difference between the test and control group depends on each operator. For operator 1, there was no significant difference between the test and control; for operator 2, the test group had, on average, a greater bone temperature difference than the control group. This may be attributed to the use of greater force or less precision in handling the saw, compromising cooling and increasing friction. Regarding the loss of bone block mass, although the variations were slight, there was a tendency for greater wear in the samples from operator 1, which may indicate lower precision and stability in performing the osteotomy. These data reinforce the importance of the learning curve and highlight the impact of technical proficiency on the safety and efficiency parameters of piezosurgery.

Finally, this study was limited to comparing two blade formats from a single manufacturer. Other variables, such as different blade dimensions, constituent materials, irrigation systems, and piezoelectric motors from different companies, were not evaluated. This limitation limits the generalizability of the results, especially in a rapidly evolving technological field. Expanding this study to multicenter comparisons, with different manufacturers and cutting-edge designs, will be essential to confirm the clinical applicability of the findings.

A universal limitation of the piezoelectric saw, regardless of the manufacturer, is its size, which is directly linked to the need to maintain a specific bone cutting frequency (24–34 KHz) and compatibility with the motor impedance [2,5,6,7]. Blade design and dimensions have always posed a technical challenge for the development and cutting performance of this technology. However, modifications to the tooth geometry open up new possibilities for the development of innovative designs and varying sizes, allowing the technology to be adapted for osteotomies in long bones, such as the femur and tibia, and regions with more complex anatomy, such as the calcaneus and pelvic ring.

The new blade’s applicability to long, dense bones expands the potential of piezosurgery beyond its traditional use in smaller bones, such as those of the face, skull, hand, and foot. This expanded scope can benefit not only experienced surgeons but also those learning, as a faster, safer technique facilitates adaptation and reduces the risk of intraoperative complications. Another disadvantage of the piezoelectric saw compared to other methods is the high cost of the blade and motor, which can be a limitation, especially in developing countries with limited resources [2,3,6,20,22].

The sample size of this study, consisting of 40 slides equally distributed between the test and control groups, represents one of this research’s strengths. Previous work in the field has often used smaller samples, which limits statistical power and increases the risk of type II error. The decision to expand the sample size aimed to ensure greater robustness in the analyses, allowing for the identification of significant differences even in discretely variable variables, such as cutting time and bone temperature. Furthermore, the inclusion of two operators, each responsible for 20 cuts, increased the external validity of the findings by bringing the experiment closer to clinical settings where technical expertise is heterogeneous. Despite this, it is recognized that studies with even larger samples, or with multiple collection centers, can provide more precise estimates and confirm the reproducibility of results in different contexts.

In summary, the results of this study demonstrate that the multidirectional geometry of the piezoelectric blade represents a significant technical advancement, providing greater cutting efficiency without compromising thermal safety. Although laboratory findings point to promising benefits, its clinical applicability still requires validation in biological models such as bovine or porcine ribs and in different surgical contexts in clinical studies. The multidirectional blade should be viewed not as an end point, but as an important step in the continuous evolution of osteotomy techniques, whose clinical impact can only be fully confirmed through future studies to validate its use in different specialties and surgical situations.

## 5. Conclusions

Given the limitations of this study, it can be concluded that the multidirectional geometry of the piezoelectric blade can improve cutting efficiency without compromising thermal safety. These results indicate the viability of the multidirectional blade as a more efficient and faster tool for ultrasonic osteotomies when compared to the straight-toothed blade.

## Figures and Tables

**Table 1:** Descriptive statistics of cutting time comparing the control group with the operator test 1, 2 and independent of the operator.

Cutting Time Results for Operator 1							
Variable	Tip Type	*n*	Average	SD	Minimum	Median	Maximum
Cutting Time (s)	Test	10	55.50	7.86	45.00	54.50	72.00
	Control	10	80.80	19.34	64.00	71.50	126.00
Cutting Time Results for Operator 2							
Variable	Tip Type	*n*	Average	SD	Minimum	Median	Maximum
Cutting Time (s)	Test	10	50.20	7.21	37.00	52.50	58.00
	Control	10	62.30	13.60	40.00	63.50	86.00
Operator-independent results—Cutting Time							
Variable	Tip Type	*n*	Average	SD	Minimum	Median	Maximum
Cutting Time (s)	Test	20	52.85	7.83	37.00	53.50	72.00
	Control	20	71.55	18.84	40.00	69.00	126.00

**Table 2:** Analysis of variance in cutting time comparing the control group with the operator-independent test.

Analysis of Variance (Ranks)—Independent of Operator					
Source	GL	SQ (Aj.)	QM (Aj.)	F Value	*p*-Value
Tip Type	1	1946	1946.03	21.90	<0.001
Error	38	3376	88.84		
Total	39	5322			

**Table 3:** Descriptive statistics of maximum bone temperature (in degrees Celsius) comparing the control group with the test.

Maximum Bone Temperature—Operator Independent							
Variable	Tip Type	*n*	Average	SD	Minimum	Median	Maximum
Max Bone Temp. (°C)	Test	20	30.45	3804	27	29	40
	Control	20	29.4	2501	27	29	39

**Table 4:** Descriptive statistics of bone temperature difference (final—Initial) (in degrees Celsius) comparing the control group with the test for Operator 1, 2 and independent of the operator (Figure 8 and Figure 9).

Bone Temperature Difference (Final—Initial) Results for Operator 1							
Variable	Tip Type	*n*	Average	SD	Minimum	Median	Maximum
Bone Temp. Diff. (°C)	Test	10	3.8	1687	1	3.5	7
	Control	10	4.8	1033	4	4.5	7
Bone Temperature Difference (Final—Initial) Results for Operator 2							
Variable	Tip Type	*n*	Average	SD	Minimum	Median	Maximum
Bone Temp. Diff. (°C)	Test	10	6.8	4.13	2	5	14
	Control	10	3.4	3.95	0	2	14
Bone Temperature Difference (Final—Initial) Operator-Independent Results							
Variable	Tip Type	*n*	Average	SD	Minimum	Median	Maximum
Bone Temp. Diff. (°C)	Test	20	5.3	3435	1	4	14
	Control	20	4.1	2.9	0	4	14

**Table 5:** Descriptive statistics of the blade temperature difference (final—Initial) (in degrees Celsius) comparing the control group with the test.

Blade Temperature Difference (Final—Initial)—Operator Independent Results							
Variable	Tip Type	*n*	Average	SD	Minimum	Median	Maximum
Blade Temp. Diff. (°C)	Test	20	−0.19	0.832	−1.4	−0.350	1
	Control	20	0.06	0.854	−1.7	0.1	1.5

## Data Availability

The original contributions presented in this study are included in the article. Further inquiries can be directed to the corresponding authors.
